# Astrocytic Regulation of Neural Circuits Underlying Behaviors

**DOI:** 10.3390/cells10020296

**Published:** 2021-02-01

**Authors:** Sun-Nyoung Hwang, Jae Seung Lee, Kain Seo, Hyosang Lee

**Affiliations:** 1 Convergence Research Advanced Centre for Olfaction, Daegu Gyeongbuk Institute of Science and Technology (DGIST), Daegu 42988, Korea; snhwang@dgist.ac.kr; 2 Department of Brain and Cognitive Sciences, DGIST, Daegu 42988, Korea; abin1002@dgist.ac.kr (J.S.L.); Kain17@dgist.ac.kr (K.S.); 3 Korea Brain Research Institute (KBRI), Daegu 41062, Korea

**Keywords:** astrocyte, neural circuit, behavior, GFAP, chemogenetics, optogenetics

## Abstract

Astrocytes, characterized by a satellite-like morphology, are the most abundant type of glia in the central nervous system. Their main functions have been thought to be limited to providing homeostatic support for neurons, but recent studies have revealed that astrocytes actually actively interact with local neural circuits and play a crucial role in information processing and generating physiological and behavioral responses. Here, we review the emerging roles of astrocytes in many brain regions, particularly by focusing on intracellular changes in astrocytes and their interactions with neurons at the molecular and neural circuit levels.

## 1. Introduction

Astrocytes are the most abundant glial cells in the central nervous system. They are typically characterized by satellite-like morphology with numerous fine processes, but they also exhibit many morphological variations [1]. A massive morphological change, such as an increase in the number, thickness, and length of the major cellular processes, is a hallmark of reactive astrogliosis, which often occurs under pathologic conditions [2]. Genes selectively expressed in astrocytes, including glial fibrillary acidic protein (GFAP), aldehyde dehydrogenase 1 family member L1 (ALDH1L1), S100 calcium-binding protein β (S100β), L-glutamate/L-aspartate transporter (GLAST), and glutamate transporter 1 (GLT-1), have been used as a set of molecular markers to identify astrocytes and have also been utilized as genetic handles to drive transgenes in an astrocyte-specific manner [3]. Recent studies have found that astrocytes are heterogeneous in gene expression and have identified genetic markers that are relatively enriched in each brain region, for example, *Islr* in the olfactory bulb; *Kcnj10, Norrin, Olig2, Lgr6,* and *Fndc5* in the cortex; *μ-crystallin* in the striatum; *Myoc* in the dorsal midbrain; and *Gdf10* in Bergmann glia of the cerebellum [4,5,6,7,8,9,10].

**Figure 1:**
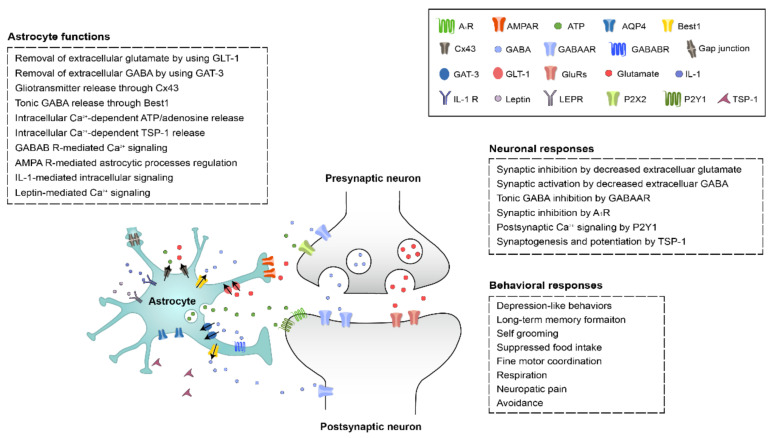
The molecular mechanism underlying astrocytic regulation of neural activity in brain regions. The detailed explanations and references can be found from the text. AA, arachidonic acid; AgRP, agouti-related peptide; AMPAR, α-amino-3-hydroxy-5-methyl-4-isoxazolepropionic acid-type glutamate receptors; AQP4, aquaporin 4; ATP, adenosine triphosphate; Best1, bestrophin 1; Cx43, connexin 43; DAO, diamine oxidase; EET, epoxyeicosatrienoic acid; GABA, γ-aminobutyric acid; GAT-3, GABA transporter type 3; GLT-1, glutamate transporter 1; IL-1, interleukin-1; LEPR, leptin receptor; MAOB, monoamine oxidase B; MSN, medium spiny neuron; PLC, phospholipase C; TC neuron, thalamocortical neuron; TSP-1, thrombospondin-1; VB, ventrobasal nucleus.

**Figure 2:**
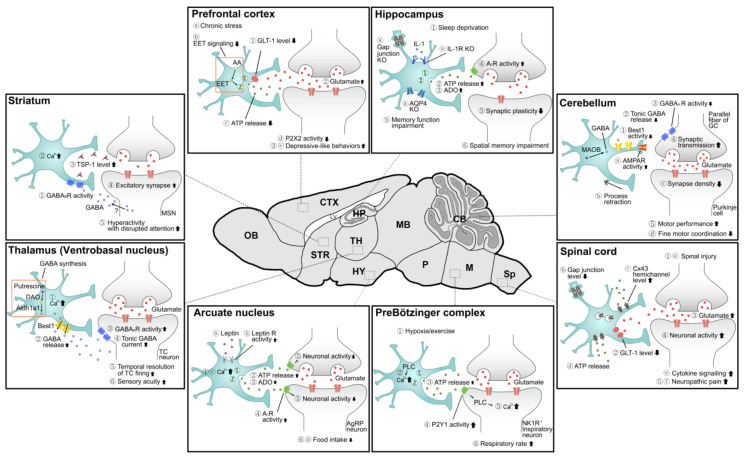
Schematic showing the intracellular signaling pathways in astrocytes and intercellular interactions between astrocytes and neurons. Separate pathways in a brain region are distinguished by numbering and alphabetical arrangement. Detailed explanations and references can be found in the text. AA, arachidonic acid; ADO, adenosine; AgRP, agouti-related peptide; AMPAR, α-amino-3-hydroxy-5-methyl-4-isoxazolepropionic acid-type glutamate receptors; AQP4, aquaporin 4; ATP, adenosine triphosphate; Best1, bestrophin 1; CB, cerebellum; CTX, cortex; Cx43, connexin 43; DAO, diamine oxidase; EET, epoxyeicosatrienoic acid; GABA, γ-aminobutyric acid; GAT-3, GABA transporter type 3; GC, granule cell; GLT-1, glutamate transporter 1; HP, hippocampus; HY, hypothalamus; IL-1, interleukin-1; LV, lateral ventricle; M, medulla; MB, midbrain; MAOB, monoamine oxidase B; MSN, medium spiny neuron; NK1R, neurokinin 1 receptor; OB, olfactory bulb; P, pons; PLC, phospholipase C; Sp, spinal cord; STR, striatum; TC neuron, thalamocortical neuron; TH, thalamus; TSP-1, thrombospondin-1.

Since astrocytes are non-excitable, they have been traditionally regarded as passive cells that support neurons in the brain [11]. For example, they supply energy sources to neurons [12], control blood flow [13], form the blood–brain barrier [14,15], and maintain extracellular ions and neurotransmitters at a proper level [16]. Astrocytes have also been found to sense the activity in a neighboring neural network through ion channels and neurotransmitter receptors in their processes and respond with an intracellular calcium transient [17,18,19,20]. Elevated intracellular calcium leads to the release of neuroactive signaling molecules, so-called gliotransmitters, from astrocytes to modulate the activity of neighboring neurons [19,20]. However, whether gliotransmission occurs under physiological conditions is controversial [21,22]. Growing evidence indicates that astrocytes participate not only in synaptic and neural circuit functions but also in various behaviors of an organism [23,24,25,26,27]. Since there are excellent reviews that summarize the molecular genetic tools developed for investigating astrocytes and some examples of their biological applications [3,28,29], we will primarily focus here on the emerging roles of astrocytes, emphasizing their interactions with neural circuits, and present recent research findings related to anatomical classification (Figure 1 and Figure 2 and Table 1 and Table 2).

## 2. Olfactory Bulb

The olfactory bulb (OB) is the brain region involved in the sense of smell [30]. There are two main types of excitatory OB neurons, the mitral cells and tufted cells (M/Ts), which relay odor information from the glomerulus to the olfactory cortex [31]. Interestingly, it has been reported that OB astrocytes adjacent to M/Ts respond to odor stimuli [32]. In vivo wide-field calcium imaging of mice expressing the genetically encoded calcium indicator GCaMP3 in M/Ts and jRCaMP1a in astrocytes has shown that M/Ts and OB astrocytes are activated in response to odorants such as isoamyl acetate, methyl salicylate, and anisole. Chemogenetic activation of the Gq pathway in OB astrocytes using hM3Dq suppresses odorant-elicited intracellular calcium in M/Ts, whereas chemogenetic stimulation of the Gi pathway in the astrocytes using hM4Di enhances the response of M/Ts. M3Dq- and hM4Di-mediated manipulations of OB astrocytes augment and lessen the odor-detection accuracy, respectively, as assessed by the Go/No-Go task. However, the molecular mechanisms underlying the interaction between astrocytes and neurons have not yet been elucidated.

## 3. Prefrontal Cortex

The prefrontal cortex (PFC) is a part of the cerebral cortex that is connected to many other brain regions, including the hippocampus, nucleus accumbens (NAc), and amygdala [33,34]. Several lines of evidence indicate that PFC astrocytes are involved in cognitive and emotional functions [25,35]. For example, pharmacological ablation of PFC astrocytes in adult rats with an astrocyte-specific toxin, L-α-aminoadipate (L-AAA), causes progressive loss and dendritic atrophy of PFC neurons and impaired memory function, as assessed by the Morris water maze and attentional set-shifting tests [35]. Ablation of PFC astrocytes also produces behavioral deficits in a battery of behavioral assays for depression and anxiety, including the sucrose preference, forced swim, active avoidance, and novelty-suppressed feeding tests [25].

One of the well-known functions of astrocytes is regulating the extracellular glutamate level, mainly by reuptake of glutamate from the synapse through the glutamate transporter GLT-1 that is expressed in their plasma membranes [36,37]. It has been shown that pharmacological intervention targeting GLT-1 in rat PFC astrocytes generates depressive-like behaviors, such as an increased threshold in intracranial self-stimulation and delayed drinking of a sucrose solution [26].

It has also been shown that astrocytes in the PFC release ATP into the extracellular space. The level of extracellular ATP in the PFC is decreased in mice susceptible to chronic social defeat stress (CSDS) but unaffected in mice resistant to this stress [24]. Intracerebroventricular injection of either ATP or ATP-γ-S, a nonhydrolyzable analog of ATP, ameliorates CSDS-evoked depressive-like behaviors, as evaluated by social interaction with conspecifics and immobility in the forced swim test (FST). These effects of ATP are abolished by systemic pretreatment with the P2 purinergic receptor antagonist suramin. A reduction in ATP release from PFC astrocytes and depressive-like behavioral phenotypes are also observed in mice genetically deleted of inositol 1,4,5-triphosphate receptor type 2 (IP_3_R2), which is the astrocyte-specific protein responsible for calcium release from intracellular stores. Similarly, astrocyte-selective overexpression of the dominant-negative domain of synaptobrevin 2 peptide (dnSNARE) in mice leads to a decreased extracellular ATP in PFC and depressive-like behaviors. On the other hand, chemogenetic activation of the Gq pathway in astrocytes by using transgenic mice overexpressing MrgprA1 will promote ATP release from PFC astrocytes and attenuate depressive-like behaviors such as immobility in FST and CSDS-induced social avoidance. Taken together, these results suggest that ATP released from PFC astrocytes exerts its antidepressant effects via the activation of P2 purinergic receptors.

A recent study has demonstrated that lipid signaling in PFC astrocytes modulates depressive-like behaviors [38]. Producing an increase in epoxyeicosatrienoic acid (EET) by injecting a mixture of inhibitors hydrolyzing EET into the PFC reduces CSDS-elicited immobility in FST, whereas the infusion of a selective antagonist of EET increases immobility in the same test. The intracellular level of EET in primary cultured astrocytes is correlated with the amount of ATP in the culture medium, suggesting that extracellular ATP mediates the anti-depressive effect of EET.

PFC astrocytes are also known to participate in the regulation of ethanol consumption and intoxication [39]. Chemogenetic activation of the Gq pathway in PFC astrocytes with hM3Dq promotes ethanol consumption and induces low-dose ethanol-evoked hyperlocomotion and the sedative effects of high-dose ethanol. In contrast, reducing intracellular calcium in PFC astrocytes by overexpressing human plasma membrane calcium ATPase isoform 2 splice variant w/b (hPMCA2w/b), a plasma membrane calcium pump, leads to a decrease in the sedative effects of high-dose ethanol, implying that astrocytic calcium signaling in PFC plays an important role in regulating biological responses following the drinking of ethanol. The prolonged hM3Dq-mediated sedative effect of high-dose ethanol is reduced by intraperitoneal administration of an A_1_ adenosine receptor antagonist, suggesting that adenosine is the astrocytic mediator evoking these behavioral consequences. Although it is still necessary to further identify the type of PFC astrocytes mediating the depressive and ethanol-elicited responses, it appears that PFC astrocytes utilize different gliotransmitters and generate different behavioral outcomes in response to different types of stimuli and contexts.

## 4. Motor and Somatosensory Cortices

It has been shown that astrocytes are necessary for motor-skill learning. Calcium imaging experiments involving brain slices prepared from mice lacking IP_3_R2 have revealed that astrocytes in the motor cortex are significantly impaired in terms of generating spontaneous and extracellular ATP-evoked intracellular calcium transients. IP_3_R2-knockout mice exhibit poor performance in the forelimb reaching task [40]. Similar electrophysiological and behavioral deficits are observed in wildtype mice receiving an intracerebroventricular injection of fluorocitrate (FC), an astrocyte-specific metabolic inhibitor. FC treatment also suppresses induction of glutamate receptor subunit 1 in the motor cortex following motor-skill learning. The effect of FC is dissipated by the intraperitoneal injection of D-serine. These results suggest that astrocytes in the motor cortex are involved in motor-skill learning that occurs via intracellular calcium signaling and D-serine release.

Astrocytes in the primary somatosensory cortex (S1) have been shown to participate in the mechanical allodynia elicited by a partial sciatic nerve ligation [41]. Following induction of nerve injury, metabotropic glutamate receptor 5 (mGluR5) signaling is enhanced in S1 astrocytes, leading to an increase in intracellular calcium transients in astrocytes and the subsequent release of synaptogenic thrombospondin 1 (TSP-1), which in turn results in the formation of new synapses in neighboring neurons and the production of mechanical allodynia.

## 5. Anterior Cingulate Cortex

The anterior cingulate cortex (ACC) is another brain region that is responsible for pain perception and accompanying emotional experiences [42,43]. It has been demonstrated that GFAP mRNA and protein expression are increased by induction of tissue inflammation in the hind paw. Ablation of ACC astrocytes by a local L-AAA injection alleviates the inflammation-induced mechanical allodynia and conditioned place avoidance [27]. There is evidence indicating that ACC astrocytes contribute to the development of neuropathic pain and related symptoms. GFAP expression in the ACC is increased after partial sciatic nerve injury [44]. The nerve injury leads to an increase in the extracellular glutamate level in the ACC, which stimulates translocation of GAT-3 to the plasma membranes to promote the importation of extracellular γ-aminobutyric acid (GABA) into astrocytes. Interestingly, neuropathic pain-induced sleep disorder can be ameliorated by injecting a GAT-3 inhibitor into the ACC [45]. Indeed, optogenetic stimulation of ACC astrocytes has been shown to elicit sleep disturbance.

## 6. Hippocampus

The hippocampus is a crucial brain structure that is responsible for learning and memory [46]. Numerous studies have been conducted to investigate the role of hippocampal astrocytes. For example, chemogenetic activation of the Gq pathway in CA1 astrocytes using hM3Dq augments the activity of neurons in the region when astrocytic activation is coupled to learning. This activation also enhances synaptic potentiation and improves spatial and contextual memory, as assessed by the T-maze and fear-conditioning tests [47]. On the other hand, activation of the Gi pathway in CA1 astrocytes using hM4Di disrupts synaptic transmission from CA3 to CA1 and inhibits the projection of CA1 neurons to the ACC. This manipulation causes an impairment in remote contextual memory in the fear conditioning test [48]. Sleep deprivation also causes deficits in synaptic plasticity in CA1 and spatial memory. These deficits are rescued by either overexpressing dnSNARE in astrocytes or by treatment of an adenosine A_1_R antagonist in the hippocampus, suggesting ATP/adenosine release from hippocampal astrocytes is responsible for sleep deprivation-induced electrophysiological and cognitive dysfunction [49].

Several astrocytic molecules in the hippocampus have been found to mediate synaptic plasticity as well as learning and memory. For example, mice deleted of interleukin-1 receptor type 1 (IL-1rKO) exhibit defective long-term potentiation (LTP) and dysfunctional memory in the fear-conditioning and Morris water maze tests [50]. These phenotypic changes are restored by the intra-hippocampal transplantation of neural precursor cells (NPCs) derived from wildtype neonatal mice. Because an in vitro analysis in the study demonstrated that NPCs differentiate mainly into astrocytes, IL-1 signaling in astrocytes may play an essential role in hippocampal synaptic plasticity and memory functions. Another astrocytic molecule mediating hippocampal functions is the water channel aquaporin-4 (AQP4) [51]. Mice lacking AQP4 display altered hippocampal synaptic plasticity and impaired object placement memory. Finally, astrocytic gap junction proteins have been shown to be required for spatial memory and sensorimotor functions. Genetic deletion of both connexin-43 (Cx43) and connexin-30 (Cx30) leads to astrocytic swelling in CA1 and impairment of sensorimotor and spatial memory functions in the rotarod, balance beam, and object recognition assays [52].

## 7. Striatum

The striatum is a primary input area in the basal ganglia that integrates excitatory and inhibitory signals from several brain areas involved in action selection, motor function, and repetitive and habitual behaviors [53,54]. A recent study has demonstrated that striatal astrocytes are strongly associated with obsessive-compulsive behaviors [55]. Reducing calcium signaling in striatal astrocytes by injecting an adeno-associated virus expressing hPMCA2w/b leads to excessive self-grooming as a result of a facilitated uptake of extracellular GABA into astrocytes through GAT-3 and a consequent decrease in the activity of GABAergic medium spiny neurons (MSNs). Striatal astrocytes also contribute to behavioral hyperactivity accompanied by disrupted attention [56]. Chemogenetic activation of the Gi pathway in astrocytes using hM4Di to mimic the activation of the Gi-coupled GABA_B_ receptor by GABA released from MSNs is sufficient to elicit an intracellular calcium transient and upregulation of TSP-1 in astrocytes, resulting in augmentation of firing of MSNs and induction of hyperlocomotion.

The nucleus accumbens (NAc) of the striatum is a brain region associated with reward and addiction that receives dopaminergic input from the ventral tegmental area (VTA) in the midbrain [57]. A recent study has demonstrated that astrocytes in the NAc respond to synaptic dopamine through D1 receptors [58]. Optogenetic activation of the nerve endings of VTA dopaminergic neurons causes intracellular calcium transients in NAc astrocytes, which are further augmented by the systemic administration of amphetamine. Astrocytic calcium elevation produced by dopamine leads to ATP/adenosine release, which then activates A_1_ adenosine receptors of presynaptic neurons in the NAc and depresses excitatory synaptic transmission to MSNs. This synaptic depression can be phenocopied by elevating intracellular calcium in astrocytes with hM3Dq. Genetic deletion of IP_3_R2 abolishes dopamine-induced calcium transients in NAc astrocytes, impairs synaptic depression in the NAc, and reduces amphetamine-induced hyperlocomotion. These findings suggest that astrocytes are the key component in the dopaminergic signaling and mesolimbic reward system.

## 8. Amygdala

The amygdala is a neural structure associated with the regulation of emotions, including fear and anxiety [59,60]. It consists of multiple functionally distinct nuclei, including the basolateral amygdala (BLA) and the central nucleus of the amygdala (CeA) [61]. The BLA is the main input structure of the amygdala, receiving diverse sensory information from the thalamus and cortex [62,63]. The CeA contains mainly GABAergic neurons and can be further divided into the lateral (CeL) and medial (CeM) subdivisions [61]. The CeM receives excitatory and inhibitory inputs from the BLA and CeL, respectively, and is the primary output region in the amygdala sending signals to other brain regions such as the brainstem and hypothalamus that are important for processing emotional information [64,65,66].

Neuroimaging studies have revealed that patients with mood disorders exhibit a volumetric reduction in the amygdala [67,68,69,70]. The size change occurs as the result of a reduction in astrocytes, and not a loss of neurons [71,72]. Preclinical studies have provided evidence showing the importance of amygdaloid astrocytes in processing emotional information. For example, an intracellular calcium increase in CeM astrocytes, achieved either through chemogenetic activation using hM3Dq or local production of endocannabinoids, leads to the release of ATP/adenosine from astrocytes and a decrease in CeM neuronal firing as a result of a suppression of excitatory synaptic transmission from the BLA to the CeM and the promotion of inhibitory synaptic transmission within the CeA, mediated via A_1_ and A_2A_ adenosine receptors [73]. Consequently, fear behavior has shown to be reduced in the delayed fear conditioning paradigm.

A molecular study identified GLT-1 in the CeA as a protein responsible for the comorbidity of depression and anxiety [74]. Inhibition of GLT-1 by local treatment of the GLT-1 inhibitor generates depressive- and anxiety-like behaviors, as indicated by the results of the intracranial self-stimulation and the elevated plus maze and fear conditioning tests. Since GLT-1 is selectively expressed in astrocytes, these comorbid changes in response to GLT-1 inhibition may be the result of impaired glutamate uptake into astrocytes via GLT-1.

In many brain regions, astrocytes are connected to each other through gap junctions. The importance of gap junctions in fear memory consolidation has been examined in the BLA [75]. Contextual freezing observed 24 h after auditory fear conditioning is dissipated by a local infusion of a Cx43 inhibitor into the BLA. The effect of Cx43 inhibition is dissipated by cotreatment with a mixture of putative gliotransmitters consisting of glutamate, glutamine, lactate, D-serine, glycine, and ATP, indicating that astrocytic Cx43 is required for gliotransmitters to perform their roles in the BLA.

Another study has found that L-lactate acts in the BLA as a gliotransmitter that is critical for drug memory reconsolidation [76]. Blocking the release of glycogen-derived L-lactate from BLA astrocytes with a local infusion of a glycogen phosphorylase inhibitor is sufficient to impair the cocaine-induced conditioned place preference. This inhibitory effect is relieved by cotreatment with L-lactate. 

One of the Rho family of GTPases, Ras-related C3 botulinum toxin substrate 1 (Rac1), is another astrocytic protein in the BLA that is involved in conditioned fear memory [77]. Animals who have experienced fear conditioning exhibit a reduction in Rac1 activity in BLA astrocytes. Activation and inhibition of astrocytic Rac1 in the BLA hampers and facilitates the acquisition of fear memory, respectively. However, it is unclear how astrocytic signaling in the amygdala is initiated in vivo.

## 9. Thalamus

Astrocytes in the ventrobasal complex (VB) and the lateral habenula (LHb) have been found to regulate somatosensation and depression, respectively [78,79]. The VB serves as a relay station, transferring sensory information from the periphery and other brain regions to the cortex, including the somatosensory cortex [80,81,82]. Tactile discrimination has been shown to be improved when thalamocortical neurons are inhibited by extracellular GABA, which is synthesized by diamine oxidase and aldehyde dehydrogenase 1a1 in VB astrocytes and exported to the extracellular space through bestrophin 1 [78].

It has been found that the rat model of depression induced by an intraperitoneal injection of lipopolysaccharide promotes the expression of inwardly rectifying potassium channels 4.1 (Kir4.1) in LHb astrocytes. Kir4.1 is found in the astrocytic plasma membrane surrounding the neuronal soma. Overexpression of Kir4.1 in LHb astrocytes decreases the resting membrane potential in LHb neurons and increases the percentage of neurons exhibiting burst firing. Mice that overexpress Kir4.1 in LHb display depression-like behaviors in the forced swim and sucrose preference tests. In contrast, knockdown of Kir4.1 in the LHb with short hairpin RNA (shRNA) produces the opposite effect on the resting membrane potential and neuronal bursting and reduces depressive-like behaviors exhibited in the congenital learned helplessness model of rats [79]. These results suggest that Kir4.1 in LHb astrocytes plays a critical role in generating depression-like behaviors.

## 10. Hypothalamus

### 10.1. Arcuate Nucleus

The arcuate nucleus of the hypothalamus (ARH) is a key region controlling appetite and energy metabolism [83]. Genetic deletion of leptin receptors leads to morphological changes in ARH astrocytes, including a decrease in the number and length of primary astrocytic projections without affecting the total number of GFAP-positive cells [84]. Moreover, it alters the number of synapses on both proopiomelanocortin (POMC) and agouti-related protein (AGRP) neurons. Behaviorally, mice lacking leptin receptors exhibit diminished leptin-mediated feeding suppression and an augmented fasting- or ghrelin-induced food intake.

Yang [85] has shown that activation of the astrocytic Gq pathway in the medial basal hypothalamus with hM3Dq suppresses ghrelin-evoked feeding but enhances leptin-induced inhibition of food intake. Conversely, the use of hM4Di to activate the astrocytic Gi pathway in the same region augments ghrelin-evoked food intake and diminishes leptin-induced anorexia. Local infusion of an adenosine A_1_ receptor antagonist increases ghrelin-evoked food intake and abolishes astrocyte activation-induced inhibition of AGRP neuronal firing. These results suggest that extracellular adenosine converted from extracellular ATP released from astrocytes regulates the activity of AGRP neurons through adenosine A_1_ receptors and feeding behavior. ARH astrocytes are also involved in high-fat diet-evoked hypothalamic inflammation and weight gain [86]. Astrocyte-specific deletion of inhibitory kappa B kinase β, a serine kinase essential for activating nuclear factor kappa B (NF-κB), has been shown to suppress astrogliosis and inflammatory reactions in the hypothalamus as well as weight gain following chronic consumption of a high-fat diet.

### 10.2. Suprachiasmatic Nucleus

The suprachiasmatic nucleus (SCN) of the hypothalamus is the central pacemaker responsible for controlling circadian rhythms [87]. Circadian rhythms have been known to be regulated by negative feedback loops of multiple clock genes at both the transcription and translation levels in SCN neurons. The key clock genes are brain and muscle Arnt-like protein-1 (Bmal1), Clock, Cryptochrome (Cry), and Period [88]. Interestingly, SCN astrocytes also express some of the clock genes. Astrocyte-specific deletion of Bmal1 in mice disrupts the circadian rhythms of SCN neurons and circadian wheel-running activity [89]. Molecular analysis has revealed that astrocytic deletion of Bmal1 leads to a reduction in the astrocytic GABA transporters GAT1 and GAT3, causing insufficient clearance of extracellular GABA released by neurons and affecting not only the circadian oscillation of the clock genes in neurons but also wheel-running activity and cognitive function [90]. These alterations are rescued by systemic treatment with a GABA_A_ antagonist, either pentylenetetrazole or picrotoxin, suggesting that Bmal1 in SCN astrocytes controls the circadian rhythm of SCN neurons and circadian behavior via GABA_A_ receptor-mediated signaling. 

Another piece of evidence showing the involvement of SCN astrocytes in circadian rhythm has been provided by a study using mice lacking both Cry1 and Cry2 [91]. The deletion of Cry1 and Cry2 from the whole body results in the absence of a circadian oscillation of extracellular glutamate and locomotive behavior that is rescued by tissue-specific genetic complementation of Cry1 in SCN astrocytes. Typically, the circadian oscillation of extracellular glutamate is synchronous with the calcium oscillation in astrocytes and is antiphase with that in neurons. Astrocyte-released glutamate binds to NR2C-containing N-methyl-D-aspartate (NMDA) receptors in the presynaptic terminals of GABAergic neurons, thereby suppressing neuronal activity in the SCN [92]. It has also been shown that circadian oscillations of glutamate and clock genes require Cx43. Taken together, these results suggest that glutamate released by SCN astrocytes is an essential mediator of circadian rhythm.

## 11. Midbrain

### 11.1. Ventral Tegmental Area

The VTA is a midbrain structure that has been implicated in reward and aversion and contains diverse neuronal cell types, including dopaminergic and GABAergic neurons [93,94]. Optogenetic stimulation of VTA astrocytes with channelrhodopsin-2 (ChR2) has been found to accumulate extracellular glutamate, which promotes the activation of GABAergic neurons that control postsynaptic dopaminergic neurons in the VTA and generates avoidance behavior in the conditioned place aversion test [95]. Genetic deletion of GLT-1 in VTA astrocytes abolishes the optogenetically elicited electrophysiological changes and avoidance behavior, while leaving the drug-seeking behavior unaffected. Thus, these findings suggest that VTA astrocytes selectively mediate aversive learning by activating GABAergic neurons in a GLT-1-dependent manner.

### 11.2. Periaqueductal Grey

The periaqueductal gray (PAG) is a midbrain region surrounding the cerebral aqueduct that receives nociceptive inputs from the spinal cord and some regions of the brain and then transmits the information to other brain regions associated with pain processing [96]. Reactive astrocytes, characterized by hypertrophic cell bodies and increased GFAP immunoreactivity, have been found in the PAG after sciatic nerve injury caused by chronic constriction (CCI) [97]. The PAG treatment of FC attenuates CCI-induced mechanical allodynia and thermal hyperalgesia, indicating that astrocytes are necessary for enhanced nociceptive responses after nerve injury. 

Reactive astrocytes have also been observed in the PAG of mice with bone cancer pain (BCP) [98]. A local infusion of L-AAA in PAG reverses BCP-induced astrocyte activation and alleviates mechanical allodynia induced by the inoculation of tumor cells into the bones. The phosphorylation of c-Jun N-terminal kinase (pJNK) is increased in the PAG by bone cancer, and an infusion of a pJNK inhibitor into the PAG suppresses astrocyte activation and mechanical allodynia accompanied by BCP, indicating that pJNK in astrocytes contributes to BCP. Since inhibitors of cytokine signaling molecules such as phosphorylated NF-κB, chemokine CXC motif ligand 1 (CXCL1), and CXC chemokine receptor 2 (CXCR2) ameliorate the symptoms of BCP, it has been proposed that BCP induces activation of NF-κB in PAG astrocytes and the release of CXCL1, which then activates CXCR2 in the PAG neurons and elicits pain hypersensitivity [99]. Altogether, these results highlight the role of PAG astrocytes in pain modulation.

## 12. Medulla Oblongata

The preBötzinger complex (preBötC) is a region of the ventrolateral medulla oblongata that is responsible for the generation of respiratory rhythm [100,101,102,103]. Astrocytes in the preBötC have been shown to play an essential role in this generation of respiratory rhythm [104,105,106,107]. Astrocytes exhibit autonomous intracellular calcium oscillation, which is unaffected by a voltage-gated sodium channel blocker, tetrodotoxin, indicating that astrocytic activity is not controlled by neural activity in the region. Optogenetic stimulation of the preBötC astrocytes using ChR2 elicits the firing of neighboring respiratory neurons. Blockade of astrocytic metabolism by either fluoroacetate or methionine sulfoximine suppresses respiratory activity [108,109]. ATP has been found to be the key mediator released by astrocytes during the process. Overexpression of an ectonucleotidase hydrolyzing extracellular ATP to adenosine diphosphate and adenosine causes a significant reduction in the resting breathing frequency [110]. A decrease in the resting respiratory rate is also observed when vesicular release from preBötC astrocytes is disrupted by overexpressing dnSNARE or exocytosis-inhibiting tetanus toxin light chain. Chemogenetic activation of preBötC astrocytes using hM3Dq increases the resting respiratory rate, and this increase is abolished by the overexpression of ectonucleotidase. Extracellular ATP has been shown to bind to metabotropic purinergic receptors (P2Y1) in preBötC neurons [111,112,113]. These results imply that preBötC astrocytes modulate the respiratory frequency via the vesicular release of ATP and subsequent activation of P2Y1R in the preBötC neurons.

## 13. Cerebellum

The cerebellum (CB) is a brain region involved in movement and coordination, including maintaining balance, coordinating movement, and motor learning [114,115]. The CB contains specialized astrocytes called Bergmann glia (BG). The cell bodies of the BG surround the Purkinje cell somata and extend their processes to enwrap the synapses on the Purkinje cell dendrites [116,117,118,119]. It has been shown that α-amino-3-hydroxy-5-methyl-4-isoxazolepropionic acid (AMPA)-type glutamate receptors (AMPARs) in the BG play a critical role in fine motor coordination [120]. For example, mice with conditional double-knockout of GluA1 and GluA4 AMPAR subunits display not only a retraction of their BG processes from Purkinje cell spines but also impair motor coordination in the Erasmus ladder and Pavlovian eyeblink conditioning tests. 

The role of astrocytic GABA in regulating motor function has also been investigated by blocking tonic GABA release from BG by using knockout mice deleted of either bestrophin 1 or astrocytic GABA synthesizing monoamine oxidase B (MAOB), or by performing pharmacological inhibition of MAOB with selegiline [121]. Such manipulations turn out to enhance the excitability of cerebellar granule cells (GCs) and motor coordination in the rotarod test. Facilitation of tonic GABA release by overexpressing MAOB in astrocytes results in the opposite results in terms of electrophysiological and behavioral responses. Therefore, it can be concluded that astrocytic tonic GABA synthesized by MAOB and released through bestrophin 1 inhibits GCs and impairs motor performance.

## 14. Spinal Cord

The potential involvement of astrocytes in chronic itch has been identified in the spinal dorsal horn, a key region involved in the processing and relaying of itch signals received from pruriceptors to the brain [122,123]. Reactive astrogliosis is observed in the spinal dorsal horn of mouse models of chronic itch, and this astrogliosis has been found to be dependent on TRPV1-expressing primary sensory neurons. Signal transducer and activator of transcription 3 (STAT3) in astrocytes has been identified as a critical player in this process since genetic deletion or pharmacological inhibition of STAT3 alleviates astrogliosis in the spinal cord but also reduces chronic itch-evoked scratching. Lipocalin 2 generated by reactive astrocytes has been proposed to act as an extracellular signaling molecule.

Multiple lines of evidence indicate that astrocytes in the spinal cord participate in pain processing, particularly that of chronic pain. For example, selective genetic deletion of GLT-1 only from astrocytes in the spinal cord results in aggravated tactile allodynia in the mouse model of neuropathic pain [124]. Interestingly, these mice exhibit normal responses to mechanical and thermal stimuli in the absence of injury, suggesting that GLT-1 is selectively involved in chronic pain. Chronic treatment with ceftriaxone, an antibiotic exerting an analgesic effect on neuropathic pain, increases GLT-1 expression in the spinal cord [125]. Such an analgesic effect of ceftriaxone is absent from mice deleted of GLT-1 in the astrocytes of their spinal cords, suggesting that the pain relief by ceftriaxone requires GLT-1.

Nerve injury causes upregulation of Cx43 and also a transition from Cx43-containing gap junctions to Cx43 hemichannels in astrocytes [27]. Astrocyte-specific deletion of Cx43 and Cx30 reduces not only the levels of extracellular ATP and reactive astrocytes in the spinal cord but also the mechanical allodynia and heat hyperalgesia that accompany nerve injury [27,126,127]. It has been suggested that a release of ATP through astrocytic Cx43 hemichannels promotes nociception during chronic pain.

Finally, Nam and coworkers have demonstrated that optogenetic manipulation of astrocytes in the spinal cord’s dorsal horn using ChR2 leads to ATP release [128]. Extracellular ATP inhibits GABAergic interneurons in the dorsal horn by activating adenosine A_1_ receptors and therefore disinhibiting NK1R-expressing projection neurons. In behavioral terms, optogenetic stimulation of astrocytes is sufficient to elicit mechanical allodynia and thermal hyperalgesia in uninjured animals and augments nocifensive behavior in animals with neuropathic pain.

## 15. Concluding Remarks

Many recent studies have identified astrocytes as one of the pivotal components required for information processing in the central nervous system and the generation of physiological and pathological behaviors (Table 1 and Table 2). The novel role of astrocytes has been mainly revealed by studies adapting astrocyte-specific promoters and markers, such as GFAP, ALDH1L1, S100b, GLAST, and GLT-1, in combination with modern molecular genetic tools, to selectively label, monitor, and manipulate astrocytes in vivo [3,5]. These studies aim to cover as many astrocytes as possible in a target brain region, while leaving neurons unaffected. One of the critical questions that remains to be addressed is whether astrocytes in a particular brain region are functionally homogeneous or are composed of distinct subpopulations. RNA sequencing studies at the single-cell and brain region levels have demonstrated that astrocytes are molecularly heterogeneous and can be classified into several subtypes according to their gene expression profiles [8,129,130,131,132]. Although molecular diversity does not necessarily mean functional distinction, future studies may further divide the astrocytes into subpopulations and manipulate individual subpopulations in order to examine the behavioral consequences of their manipulation. Simultaneously, we have to consider that some astrocytes are connected via gap junctions and appear to function in coordination [133]. Thereby, intracellular changes in a cell such as calcium elevation can be spread to the entire astrocytic network.

A brain region typically contains heterogeneous subpopulations of neurons that contribute to generating a behavioral outcome through excitatory and inhibitory interactions. Many brain regions have also been found to contain subpopulations of neurons that mediate distinct or opposing behaviors [134,135,136,137,138,139,140,141,142,143]. Because neurons and astrocytes are often intermingled in a brain area, it is unclear how experimental manipulation of astrocytes in the whole area generates responses from specific sets of neurons and, consequently, a behavior. Several lines of evidence have shown that such specificity can be achieved in part by restricting the expression of receptors and proteins that respond to astrocyte-driven signaling molecules in a subpopulation of neurons [20,144,145,146,147,148]. Interestingly, different neural responses are elicited by the same extracellular signaling molecules, depending on the neurons and brain regions involved [24,58,73,78,84,121]. Future studies may have to further identify and establish the molecular mechanism by which astrocytic information flows through a specific population of neurons. 

One of the barriers to understating the role of astrocytes is the difficulty in determining the inputs that initiate astrocytic responses in vivo. Unlike the situation in neurons, physical or chemical interaction of astrocytes with potential communicating partners such as neurons and microglia is difficult to identify. Some studies have shown that astrocytes are activated either by neural signals passing through the neighboring neurons or by inflammation and pathological changes in the brain area [27,95,97,122,149,150,151]. However, other studies have only presented the cellular and behavioral consequences of chemogenetic and optogenetic manipulations of astrocytes, with no interpretation of the physiological relevance of the manipulation. 

Another obstacle to studying astrocytes in vivo is the limited efficacy of molecular genetic tools to perform so-called “loss-of-function” manipulations. Except for cytotoxic chemicals ablating astrocytes, most of the available tools are designed to inhibit an elevation of calcium in the cytosolic compartment of astrocytes, which is the key event necessary for astrocytic function [152,153]. For example, several strategies have been developed to block intracellular signaling pathways that lead to calcium release from the endoplasmic reticulum or to eliminating calcium from the cytosolic compartment by pumping it out of cells. Mice lacking IP_3_ receptor type 2, which is the most abundant isoform of IP_3_R in astrocytes, have been employed in many studies and contributed significantly to revealing novel functions of astrocytes from diverse brain regions [24,154,155]. Other studies, however, have questioned the efficacy of this mouse model by pointing out the potential complications stemming from eliminating IP_3_R2 from the whole body throughout the lifetime of the animal and the insufficient inhibition of calcium signaling in astrocytes [55,154,156,157]. A few recent studies have devised alternative methods to block IP_3_R-mediated calcium release in astrocytes by overexpressing either the IP_3_ ligand-binding domain of IP_3_R1 or the pleckstrin homology domain of phospholipase C-like protein p130 to compete with the endogenous IP_3_Rs for IP_3_ binding [158,159]. 

The chemogenetic and optogenetic toolbox primarily developed to study neurons has also been introduced for the study of astrocytes [47,48,56,160,161]. Some tools, such as the chemogenetic activator hM3Dq, effectively stimulate astrocytes, whereas others, such as the chemogenetic silencer hM4Di, have generated inconsistent results, depending on the study and the target brain regions involved. In neurons, the Gi-coupled receptor hM4Di promotes activation of G protein inwardly rectifying potassium (GIRK) channels via Gβγ subunits, thereby releasing potassium from cells and resulting in the hyperpolarization of neurons [162]. In contrast, hM4Di in astrocytes appears to stimulate different signaling pathways by a mechanism that has not yet been well established. As such, some studies have demonstrated that hM4Di can promote intracellular calcium increase in astrocytes, as does hM3Dq, but other studies have reported that hM4Di causes an “inhibitory” effect in astrocytes and leads to a behavioral phenotype opposite to that obtained with hM3Dq-mediated manipulation [32,48,56]. Thus, the consequences of hM4Di-mediated manipulation should be carefully interpreted. An alternative approach to controlling intracellular calcium is overexpressing hPMCA2w/b in astrocytes [55]. hPMCA2w/b constitutively pumps intracellular calcium out of cells at the expense of ATP. Two previous studies have demonstrated that overexpression of hPMCA2w/b in astrocytes by using an adeno-associated viral system not only inhibits the intracellular calcium rise but also affects the behavioral response [39,55]. Neither the strategies targeting IP_3_R nor those involving hPMCA2w/b allow for reversible, temporally controlled manipulation, which is required for establishing a causal relationship between a manipulation and its behavioral consequences. ChR2, the most widely used optogenetic tool, is a nonselective cation channel gated by blue light. When expressed in neurons, the opening of ChR2 causes a passive diffusion of proton, sodium, potassium, and calcium ions down their concentration gradients, which leads to depolarization of the plasma membrane and the generation of action potentials. ChR2 has been adapted in the study of astrocytes often as a “gain-of-function” tool. A recent study has shown an unanticipated finding that ChR2 in astrocytes can lead to activation of nearby neurons by elevating extracellular potassium ions [163]. Although advanced molecular genetic tools have overcome the major limitations of conventional pharmacological interventions, such as off-target effects, insufficient spatiotemporal control of drug treatment, and difficulties in histological verification of drug effects, the physiological relevance of research findings obtained using optogenetic and chemogenetic manipulations should be carefully assessed. Furthermore, additional tools and further technological advances are necessary if we are to comprehensively decipher the role of astrocytes.

In vivo and ex vivo electrophysiological recording and imaging have contributed significantly to revealing neural circuits underlying behavior by establishing the correlative relationship between neural activity and behavioral outcome. They have also disclosed the molecular mechanisms regulating neural activity and synaptic transmission. However, these pieces of evidence are often lacking in the study of astrocytes and are frequently replaced by a surrogate measurement of subsequent neural activity. Recently, there have been a few studies designed to obtain electrophysiological recordings of both astrocytes and neurons in order to reveal the mechanism(s) underlying the interactions between neurons and glia at the molecular level. For example, Woo et al. found two kinetically separable glutamate release mechanisms in astrocytes by using cerebellar slice recordings [164]. Following activation of G protein-coupled receptors, astrocytes quickly release glutamate through two-pore domain potassium channel TREK-1 to activate neuronal mGluRs; this quick response is followed by the slower glutamate release through bestrophin 1 that activates synaptic NMDA receptors. Ex vivo fluorescence microscopy using genetically encoded calcium indicators has been used to image astrocytic calcium responses not only in the somata but also from the processes and endfeet. This approach has identified heterogeneity in calcium signals within an astrocyte in terms of amplitude, calcium source, and compartmental responses during sensory stimulation of a subject animal [160]. Using a combinatorial approach of electrophysiology and two-photon imaging to measure extracellular glutamate in the cingulate cortex, Romanos et al. have recently demonstrated that astrocytic dysfunction is responsible for an increase in cortical dendritic excitability and cranial pain in familial migraine [165]. Taken together, these findings indicate that greater effort must be put into recording and imaging astrocytes and their signaling molecules in future studies in order to elucidate more clearly the physiology of astrocytes and mechanisms of communication between neurons and glia.

Although astrocytes are the most abundant cell type in the brain, for many years they have received very little attention when compared to the extensive studies that have been conducted on neurons. With the advent of novel experimental techniques in recent years, significant progress has been made in our understanding of astrocytes [3]. Together with their well-known role in homeostatic support, astrocytes have become an essential part of our understanding of the neural circuit mechanisms underlying information processing and behavior. Continued research efforts involving astrocytes will broaden our knowledge of the general neurobiological principles undergirding the encoding of complex information by neural circuits and should contribute to the development of novel therapeutic strategies for treating neurological and psychological disorders by targeting this cell type.

**Table 1:** Astrocyte-specific manipulation and its cellular and behavioral consequences.

Target Region	Manipulation	Change in Astrocytes	Consequence of Manipulation		Ref
			Circuit	Behavior	
OB	Aldh1L1-cre mice/ GFAP: FLEX-hM3Dq	n.d.	Odor-evoked Ca^2+^ in M/Tsș↓	Odor detection accuracy in Go/No-Go task↑	[32]
	Aldh1L1-cre mice/ GFAP: FLEX-hM4Di	n.d.	Odor-evoked Ca^2+^ in M/Ts↑	Odor detection accuracy in Go/No-Go task↓	
PFC	WT mice/L-AAA (astrocyte-specific cytotoxin)	GFAP^+^ cells↓	Progressive neuronal loss	Spatial memory in Morris water maze test↓ Reversal learning in attentional set-shifting task↓	[35]
	WT mice/L-AAA	GFAP^+^ cells↓	n.d.	Sucrose preference in SPT↓ Latency to feed in novelty suppressed feeding test↑ Immobility in FST↑ Escape latency in active avoidance test↑	[25]
	WT mice/DHK (GLT-1 inhibitor)	n.d.	n.d.	Intracranial self-stimulation threshold↑ Latency to begin drinking sucrose in sucrose intake test↑	[26]
	WT mice/ATP or ATP-γ-S	n.d.	n.d.	CSDS-induced social avoidance in SIT↓ CSDS-induced immobility in FST↓ CMS-induced fur condition↓ Post-CMS sucrose preference in SPT↑	[24]
	IP_3_R2^−/−^ mice	ATP release↓	n.d.	Immobility in FST↑ Fur condition↓ Sucrose consumption in SPT↓	
	GFAP-tTA;tetO-dnSNARE mice	ATP release↓	n.d.	Immobility in FST↑	
	GFAP-tTA;tetO-MrgprA1 mice	ATP release↑	n.d.	Immobility in FST↓ Social avoidance in SIT↓	
	WT mice/AAV GfaABC1D-hM3Dq	c-Fos^+^ cells↑	n.d.	Ethanol consumption & preference↑ Locomotor activity (by low-dose ethanol)↑ Duration of LORR (by high-dose ethanol)↑	[39]
	WT mice/AAV GfaABC1D::hPMCA2w/b	c-Fos^+^ cells↓	n.d.	Ethanol consumption, preference↓ Duration of LORR (high-dose ethanol)↓	
MC	GLAST-creER;IP_3_R2^fl/fl^ mice	Ca^2+^↓	n.d.	Motor-skill learning in forelimb reaching task↓	[40]
	WT mice/fluorocitrate (astrocyte metabolic inhibitor)	Ca^2+^↓	LTP↓, GluA1↓	Motor-skill learning in forelimb reaching task↓	
SC	IP_3_R2^−/−^ mice	Ca^2+^↓	PSL-induced TSP-1 upregulation↓	PSL-induced mechanical allodynia↓	[41]
	WT mice/BAPTA-AM (Ca^2+^ chelator)	n.d.		PSL-induced mechanical allodynia↓	
	Fluoroacetate (astrocyte metabolic inhibitor)	n.d.	n.d.	PSL-induced mechanical allodynia↓	
ACC	L-AAA	GFAP intensity↓	n.d.	CFA-induced place escape/avoidance↓	[27]
	GFAP-cre mice/ AAV CBA::FLEX-ChR2	n.d.	n.d.	Non-rapid eye movement sleep↓	[44]
	WT mice/SNAP-5114 (GAT-3 inhibitor)	GAT-3 activity↓	n.d.	PSL-induced sleep disturbance↓	[45]
HP	WT mice/AAV GFAP::hM3Dq	Ca^2+^↑	LTP↑ Neuronal c-Fos in CA1 (when learning is combined)↑	Spatial memory in T-maze test↑ Contextual memory in fear conditioning test↑	[47]
	WT mice/AAV GFAP::hM4Di	Ca^2+^↓	CA3→CA1 synaptic transmission↓ CA1→ACC neuronal activity↓	Remote contextual memory in fear conditioning test↓	[48]
	IL-1r^−/−^ mice	n.d.	LTP↓	Spatial memory in Morris water maze test↓ Contextual memory in fear conditioning test↓	[50]
	IL-1r^−/−^ mice/transplantation of WT NPCs	n.d.	LTP↑	Spatial memory in Morris water maze test↑ Contextual memory in fear conditioning test↑	
	GFAP-tTA;tetO-dnSNARE mice	n.d.	Sleep deprivation-induced LTP deficits↓	Sleep deprivation-induced spatial memory impairment in SOR test↓	[49]
	WT mice/CPT (adenosine A_1_Rantagonist)	n.d.	Sleep deprivation-induced LTP deficits↓	Sleep deprivation-induced spatial memory impairment in SOR test↓	
	AQP4^−/−^ mice	n.d.	LTP↓ LTD↓	Object recognition memory in object placement test↓	[166]
	GFAP-cre;Cx43^fl/fl^;Cx30^−/−^ mice	Edema, vacuolation	Widespread edema, vacuolation (white matter)	Spatial memory in SOR test↓ Motor coordination in rotarod and balance beam assays↓	[52]
STR	WT mice/AAV GfaABC1D::hPMCA2w/b	Ca^2+^↓ GAT-3↑	Tonic GABA current↓ D1-MSN tonic inhibition↓ MSN excitability↓	Duration of self-grooming↑ Travel distance in open field test↓ Time spent in center in open field test↓	[55]
	WT mice/AAV GfaABC1D::hM4Di	Ca^2+^↑ TSP-1↑	MSN EPSC↑ MSN firing↑	Travel distance in open field test↑ Investigatory activity in light/dark open field test↓ Exploration of novel object in NOR test↓	[56]
	DRD1^fl/fl^ mice/AAV GFAP::mCherry-cre	DA-evoked Ca^2+^↓	DA-evoked synaptic depression↓	Amphetamine-evoked locomotion enhancement↓	[58]
	IP_3_R2^−/−^ mice	DA-evoked Ca^2+^↓	DA-evoked synaptic depression↓	Amphetamine-evoked locomotion enhancement↓	
	WT mice/AAV GFAP::hM3Dq	Intracellular Ca^2+^↑ ATP/adenosine release↑	Synaptic depression↑	n.d.	
AMY	WT mice/AAV GFAP::hM3Dq	Intracellular Ca^2+^↑	BLA→CeM excitatory synaptic transmission↓(by A_1_R↑) CeL→CeM inhibitory synaptic transmission↑(by A_2_R↑) CeM neuronal firing↓	Fear memory formation in fear conditioning tes↓	[73]
	WT mice/DHK (GLT-1 inhibitor)	n.d.	n.d.	Intracranial self-stimulation threshold↑ Time spent in open arms in elevated plus maze test↓ Freezing behavior in fear conditioning test↑	[74]
	WT mice/TAT-Cx43L2 (Cx43-hemichannel blocker)	Cx43 activity↓	No effects on synaptic release	Fear memory consolidation in fear conditioning test↓	[75]
VB	WT mice/AAV Aldh1l1::cre and AAV pSico::DAO shRNA or AAV pSico::Aldh1a1 shRNA or AAV pSico::Best1 shRNA	Tonic GABA release↓	Tonic GABA current↓ Temporal resolution↓	Tactile discrimination in tactile-based NOR test↓	[78]
	THIP (GABA_A_R agonist)	Intracellular Ca^2+^↑ Tonic GABA release↑	Tonic GABA current↑ Temporal resolution of stimulation-evoked TC neuronal firing↑	Tactile discrimination in tactile-based NOR test↑	
LHb	WT rats/AAV GfaABC1D::Kir4.1	RMP↓	Extracellular K^+^↓ Neuronal RMP↓ Neuronal bursting↑	Immobility in FST↑ Sucrose preference in SPT↓	[79]
	WT rats/AAV H1::Kir4.1-shRNA or AAV GfaABC1D::dnKir4.1		Neuronal bursting↓	Immobility in FST↓ Bar pressing in learned helplessness test↑ Sucrose preference in SPT↑	
ARH	GFAP-creERT2;Lepr^fl/fl^ mice	Number and length of projection↓	Number of synapses onto POMC neurons↑ Glial coverage on the perikaryal membranes of POMC↓ mIPSC in POMC and AgRP neurons↑ mEPSC in AgRP neurons↑	Leptin-induced suppression of food intake↓ Fasting- and ghrelin-induced food intake↑	[84]
	WT mice/AAV GFAP::hM3Dq	n.d.	Activity of AgRP neurons↓	Ghrelin-induced food intake↓ Leptin-induced suppression of food intake↓	[85]
	WT mice/AAV GFAP::hM4Di	n.d.	n.d.	Ghrelin-induced food intake↑ Leptin-induced suppression of food intake↓	
	WT mice/DPCPX (adenosine A_1_R antagonist)	n.d.	Activity of AgRP neurons↑	Ghrelin-induced food intake↑	
SCN	Aldh1L1-cre;Bmal1^fl/fl^ mice	n.d.	Altered circadian oscillation	Altered circadian wheel-running activity	[89]
	GLAST-creERT2;Bmal1^fl/fl^ mice	GAT-1, GAT-3↓	Extracellular GABA level↑ Altered circadian oscillations of clock genes in neurons	Altered circadian wheel-running activity Cognitive deficits in NOR and SOL tests↑	[90]
	Cry1/2^−/−^ mice/AAV GFAP::cre + AAV Cry1:: FLEX-Cry1	Recovery of TTFL	Recovery of circadian oscillation of extracellular glutamate	Recovery of circadian locomotor activity	[91]
VTA	WT mice/AAV GfaABC1D::ChR2	Na^+^, H^+^↑ K^+^↓	GABAergic neuronal activity↑ Dopaminergic neuronal activity↓	Real-time avoidance behavior in RTPP assay↑ Learned avoidance behavior in CPA assay↑	[95]
	GLT-1^fl/fl^ mice/ AAV GfaABC1D::ChR2 + AAV GfaABC1D::cre	n.d.	ChR2-induced increase in GABAergic neuronal activity↓ Dopaminergic neuronal activity↑	ChR2-induced increase in learned avoidance behavior in CPA assay↓	
PAG	WT mice/L-AAA (astrocytic cytotoxin) or SP600125 (JNK inhibitor)	BCP-induced GFAP upregulation↓	n.d.	BCP-induced mechanical allodynia↓	[98]
PreBötC	WT rats/AAV sGFAP::hM3Dq	Intracellular Ca^2+^↑ Vesicle fusion events↑ ATP release↑	n.d.	Resting respiratory rate↑ Frequency of sighs↑	[110]
	WT rats/AAV sGFAP::dnSNARE or Tetanus toxin light chain (exocytosis inhibitor)	Vesicle fusion events↓	n.d.	Resting respiratory rate↓ Frequency of sighs↓	
CB	GLAST-creERT2; Gria1^fl/fl^;Gria4^fl/fl^ mice	BG process retraction	EPSC frequency of PC↓ PF-PC synapse density↓	Fine motor coordination in ELT and PECT↓	[120]
	Best1^−/−^ mice or MAOB^−/−^ mice or WT mice/selegiline (MAOB inhibitor)	Tonic GABA release↓	Excitability of GCs↑ PF-PC synaptic transmission↑	Motor coordination in rotarod test↑	[121]
	GFAP-MAOB TG mice	Tonic GABA release↑	Excitability of GCs↓ PF-PC synaptic transmission↓	Motor coordination in rotarod test↓	
Spinal cord	GFAP-cre;Stat3^fl/fl^ mice or WT mice/AG490 (JAK inhibitor)	DCP-induced GFAP upregulation↓ DCP-induced LCN2 upregulation↓	DCP-induced neuronal excitation↓	DCP-induced scratching↓	[122]
	Hoxb8-cre;GLT-1^fl/fl^ mice	n.d.	n.d.	PSL-induced mechanical allodynia↑	[124]
	WT rats/AAV GfaABC1D::ChR2	ATP release↑	Adenosine A_1_R↑ Excitability of GABAergic interneurons↓ Excitability of NK1R^+^ projection neurons↑	Mechanical allodynia↑ Thermal hyperalgesia↑ SNI-induced mechanical allodynia↑	[128]

ACC, anterior cingulate cortex; AgRP, agouti-related protein; Aldh1a1, aldehyde dehydrogenase 1a1; AMY, amygdala; ARH, arcuate nucleus of hypothalamus; ATP-γ-S, adenosine 5′-(3-thiotriphosphate) tetralithium salt; BAPTA-AM, 1,2-bis(o-aminophenoxy)ethane-N,N,N’,N’-tetraacetic acid; BCP, bone cancer pain; Best1, bestrophin 1; BG, Bergmann glial cells; BLA, basolateral amygdala; CB, cerebellum; CeL, lateral central amygdala; CeM, medial central amygdala; CFA, complete Freund’s adjuvant; ChR2, channelrhodopsin; CMS, chronic mild stress; CPA, conditioned place avoidance; CPT, 8-cyclopentyl1,3-dimethylxanthine (adenosine A1 receptor antagonist); DA, dopamine; Cx, connexin; DAO, diamine oxidase; DCP, diphenylcyclopropenone; DHK, dihydrokainic acid; dnSNARE, dominant negative domain of synaptobrevin 2 peptide; ELT, Erasmus ladder test; FST, forced swim test; GCs, cerebellar granule cells; GLAST, L-glutamate/L-aspartate transporter; GLT-1, glutamate transporter 1; GluA1, glutamate receptor subunit 1; Gria, glutamate ionotropic receptor AMPA type; GRP, gastrin-releasing peptide; GS, glutamine synthetase; HP, hippocampus; HY, hypothalamus; IL-1r, interleukin-1 receptor type 1; IP_3_R2, inositol 1,4,5-trisphosphate receptor type 2; JAK, janus kinase; Kir, inward-rectifier K^+^ channel; L-AAA, L-α-aminoadipate; LCN2, lipocalin 2; Lepr, leptin receptor; LHb, lateral habenula; LORR, loss of righting reflex; LTD, long-term depression; LTP, long-term potentiation; MAOB, monoamine oxidase B; MC, motor cortex; mEPSC, miniature excitatory postsynaptic currents; mIPSC, miniature inhibitory postsynaptic currents; NK1R, neurokinin-1 receptor; Mrgpr, Mas-related G protein-coupled receptors; NOR, novel object recognition; NPCs, neural precursor cells; OB, olfactory bulb; PAG, periaqueductal gray; PC, Purkinje cell; PECT, Pavlovian eyeblink conditioning test; PF, parallel fiber; PFC, prefrontal cortex; POMC, proopiomelanocortin; PreBötC, preBötzinger complex; PSL, partial sciatic nerve ligation; RMP, resting membrane potential; RTPP, real-time place preference; SC, somatosensory cortex; SCN, suprachiasmatic nucleus; SIT, social interaction test; SNAP-5114, (S)-1-[2-[tris(4-methoxyphenyl) methoxy]ethyl]-3-piperidine carboxylic acid (i.e., GAT-3 inhibitor); SNI, spinal nerve injury; SOL, spatial object location; SOR, spatial object recognition; SPT, sucrose preference test; STR, striatum; TC neuron, thalamocortical neuron; THIP, 4,5,6,7-tetrahydroisoxazolo[5 ,4-c]pyridin-3-ol, TSP, thrombospondin; TTFL, transcription-translation feedback loop; VB, ventrobasal nucleus of thalamus; VTA, ventral tegmental area; WT, wildtype; n.d., not determined; →, projecting to; ↑, increase; ↓, decrease.

**Table 2:** Behavioral results of astrocyte-specific manipulation.

Behavior	Target Region	Manipulation	Major Phenotype	Ref
Cognition	PFC	WT mice/L-AAA (astrocyte-specific cytotoxin)	Spatial memory in Morris water maze test↓ Reversal learning in attentional set-shifting task↓	[35]
	ACC	GFAP-cre mice/ AAV CBA::FLEX-ChR2	Non-rapid eye movement sleep↓	[44]
	HP	WT mice/AAV GFAP::hM3Dq	Spatial memory in T-maze test↑ Contextual memory in fear conditioning test↑	[47]
		WT mice/AAV GFAP::hM4Di	Remote contextual memory in fear conditioning test↓	[48]
		IL-1r^−/−^ mice	Spatial memory in Morris water maze test↓ Contextual memory in fear conditioning test↓	[50]
		IL-1r^−/−^ mice/transplantation of WT NPCs	Spatial memory in Morris water maze test↑ Contextual memory in fear conditioning test↑	[50]
		GFAP-tTA;tetO-dnSNARE mice	Sleep deprivation-induced spatial memory impairment in SOR test↓	[49]
		WT mice/CPT (adenosine A_1_ receptor antagonist)	Sleep deprivation-induced spatial memory impairment in SOR test↓	[49]
		AQP4^−/−^ mice	Object recognition memory in object placement test↓	[166]
		GFAP-cre;Cx43^fl/fl^;Cx30^−/−^ mice	Spatial memory in SOR test↓	[52]
	AMY	WT mice/AAV GFAP::hM3Dq	Fear memory formation in fear conditioning test↓	[73]
		WT mice/DHK (GLT-1 inhibitor)	Freezing behavior in fear conditioning test↑	[74]
		WT mice/TAT-Cx43L2 (Cx43-hemichannel blocker)	Fear memory consolidation in fear conditioning test↓	[75]
	SCN	GLAST-creERT2;Bmal1^fl/fl^ mice	Altered circadian wheel-running activity Cognitive deficits in NOR and SOL tests↑	[90]
	VTA	WT mice/AAV GfaABC1D::ChR2	Real-time avoidance behavior in RTPP assay↑ Learned avoidance behavior in CPA assay↑	[95]
		GLT-1^fl/fl^ mice/ AAV GfaABC1D: ChR2 + AAV GfaABC1D: cre	ChR2-induced increase in learned avoidance behavior in CPA assay↓	[95]
Circadian rhythm	SCN	Aldh1L1-cre;Bmal1^fl/fl^ mice	Altered circadian wheel-running activity	[89]
		GLAST-creERT2;Bmal1^fl/fl^ mice	Altered circadian wheel-running activity	[90]
		Cry1/2^−/−^ mice/AAV GFAP::cre + AAV Cry1:: FLEX-Cry1	Recovery of circadian locomotor activity	[91]
Emotion	PFC	WT mice/L-AAA	Sucrose preference in SPT↓ Latency to feed in novelty suppressed feeding test↑ Immobility in FST↑ Escape latency in active avoidance test↑	[25]
		WT mice/DHK (GLT-1 inhibitor)	Intracranial self-stimulation threshold↑ Latency to begin drinking sucrose in sucrose intake test↑	[26]
		WT mice/ATP or ATP-γ-S	CSDS-induced social avoidance in SIT↓ CSDS-induced immobility in FST↓ CMS-induced fur condition↓ Post-CMS sucrose preference in SPT↑	[24]
		IP_3_R2^−/−^ mice	Immobility in FST↑ Fur condition↓ Sucrose consumption in SPT↓	[24]
		GFAP-tTA;tetO-dnSNARE mice	Immobility in FST↑	[24]
		GFAP-tTA;tetO-MrgprA1 mice	Immobility in FST↓ Social avoidance in SIT↓	[24]
	STR	WT mice/AAV GfaABC1D::hPMCA2w/b	Duration of self-grooming↑ Travel distance in open field test↓ Time spent in center in open field test↓	[55]
		WT mice/AAV GfaABC1D::hM4Di	Travel distance in open field test↑ Investigatory activity in light/dark open field test↓ Exploration of novel object in NOR test↓	[56]
	AMY	WT mice/DHK (GLT-1 inhibitor)	Intracranial self-stimulation threshold↑ Time spent in open arms in elevated plus maze test↓	[74]
	LHb	WT rats/AAV GfaABC1D::Kir4.1	Immobility in FST↑ Sucrose preference in SPT↓	[79]
		WT rats/AAV H1::Kir4.1-shRNA or AAV GfaABC1D::dnKir4.1	Immobility in FST↓ Bar pressing in learned helplessness test↑ Sucrose preference in SPT↑	[79]
Motor skill	MC	GLAST-creER;IP_3_R2^fl/fl^ mice	Motor-skill learning in forelimb reaching task↓	[40]
		WT mice/fluorocitrate (astrocyte metabolic inhibitor)	Motor-skill learning in forelimb reaching task↓	[40]
	HP	GFAP-cre;Cx43^fl/fl^;Cx30^−/−^ mice	Motor coordination in rotarod and balance beam assays↓	[52]
	CB	GLAST-creERT2;Gria1^fl/fl^;Gria4^fl/fl^ mice	Fine motor coordination in ELT and PECT↓	[120]
		Best1^−/−^ mice or MAOB^−/−^ mice or WT mice/selegiline (MAOB inhibitor)	Motor coordination in rotarod test↑	[121]
		GFAP-MAOB TG mice	Motor coordination in rotarod test↓	[121]
Sensory processing	OB	Aldh1L1-cre mice/ AAV GFAP::FLEX-hM3Dq	Odor detection accuracy in Go/No-Go task↑	[32]
		Aldh1L1-cre mice/ AAV GFAP::FLEX-hM4Di	Odor detection accuracy in Go/No-Go task↓	[32]
	SC	IP_3_R2^−/−^ mice	PSL-induced mechanical allodynia↓	[41]
		WT mice/BAPTA-AM (Ca^2+^ chelator)	PSL-induced mechanical allodynia↓	[41]
		Fluoroacetate (astrocyte metabolic inhibitor)	PSL-induced mechanical allodynia↓	[41]
	ACC	L-AAA	CFA-induced place escape/avoidance↓	[27]
		WT mice/SNAP-5114 (GAT-3 inhibitor)	PSL-induced sleep disturbance↓	[45]
	VB	WT mice/AAV Aldh1l1::cre and AAV pSico::DAO shRNA or AAV pSico::Aldh1a1 shRNA or AAV pSico::Best1 shRNA	Tactile discrimination in tactile-based NOR test↓	[78]
		THIP (GABA_A_R agonist)	Tactile discrimination in tactile-based NOR test↑	[78]
	PAG	WT mice/L-AAA (astrocytic cytotoxin) or SP600125 (JNK inhibitor)	BCP-induced mechanical allodynia↓	[98]
	Spinal cord	GFAP-cre;Stat3^fl/fl^ mice or WT mice/AG490 (JNK inhibitor)	DCP-induced scratching↓	[122]
		Hoxb8-cre;GLT-1^fl/fl^ mice	PSL-induced mechanical allodynia↑	[124]
		WT rats/AAV GfaABC1D::ChR2	Mechanical allodynia Thermal hyperalgesia SNI-induced mechanical allodynia↑	[128]
Appetite	ARH	GFAP-creERT2;Lepr^fl/fl^ mice	Leptin-induced suppression of food intake↓ Fasting- and ghrelin-induced food intake↑	[84]
		WT mice/AAV GFAP::hM3Dq	Ghrelin-induced food intake↓ Leptin-induced suppression of food intake↑	[85]
		WT mice/AAV GFAP::hM4Di	Ghrelin-induced food intake↑ Leptin-induced suppression of food intake↓	[85]
		WT mice/DPCPX (adenosine A_1_R antagonist)	Ghrelin-induced food intake↑	[85]

ACC, anterior cingulate cortex; Aldh1a1, aldehyde dehydrogenase 1a1; AMY, amygdala; ARH, arcuate nucleus of hypothalamus; AQP4, aquaporin 4; ATP-γ-S, adenosine 5′-(3-thiotriphosphate) tetralithium salt; BAPTA-AM, 1,2-bis(o-aminophenoxy)ethane-N,N,N’,N’-tetraacetic acid; BCP, bone cancer pain; Best1, bestrophin 1; Bmal1, brain and muscle Arnt-like protein-1; CB, cerebellum; CFA, complete Freund’s adjuvant; ChR2, channelrhodopsin; CMS, chronic mild stress; CPA, conditioned place avoidance; CPT, 8-cyclopentyl1,3-dimethylxanthine (adenosine A1 receptor antagonist); CSDS, chronic social defeat stress; Cx, connexin; DAO, diamine oxidase; DCP, diphenylcyclopropenone; DHK, dihydrokainic acid; dnSNARE, dominant negative domain of synaptobrevin 2 peptide; DPCPX, 8-cyclopentyl-1,3-dipropylxanthine; ELT, Erasmus ladder test; FST, forced swim test; GLAST, L-glutamate/L-aspartate transporter; GLT-1, glutamate transporter 1; HP; hippocampus; IL-1r, interleukin-1 receptor type 1; IP_3_R2, inositol 1,4,5-trisphosphate receptor type 2; JNK, c-Jun N-terminal kinase; L-AAA, L-α-aminoadipate; Lepr, leptin receptor; LHb, lateral habenula; MAOB, monoamine oxidase B; MC, motor cortex; Mrgpr, Mas-related G protein-coupled receptors; NOR, novel object recognition; NPCs, neural precursor cells; OB, olfactory bulb; PAG, periaqueductal gray; PECT, Pavlovian eyeblink conditioning test; PFC, prefrontal cortex; PSL, partial sciatic nerve ligation; RTPP, real-time place preference; SC, somatosensory cortex; SCN, suprachiasmatic nucleus; SIT, social interaction test; SNAP-5114, (S)-1-[2-[tris(4-methoxyphenyl) methoxy]ethyl]-3-piperidine carboxylic acid (i.e., GAT-3 inhibitor); SNI, spinal nerve injury; SOL, spatial object location; SOR, spatial object recognition; SPT, sucrose preference test; STR, striatum; THIP, 4,5,6,7-tetrahydroisoxazolo[5,4-c]pyridin-3-ol; VB, ventrobasal nucleus of thalamus; VTA, ventral tegmental area; WT, wildtype; ↑, increase; ↓, decrease.

## Data Availability

Not applicable.
